# The Importance of Competitiveness in New Internationalized and Competitive Environment of Pharmaceutical Industry

**Published:** 2014

**Authors:** Gholamhossein Mehralian, Hosein Shabaninejad


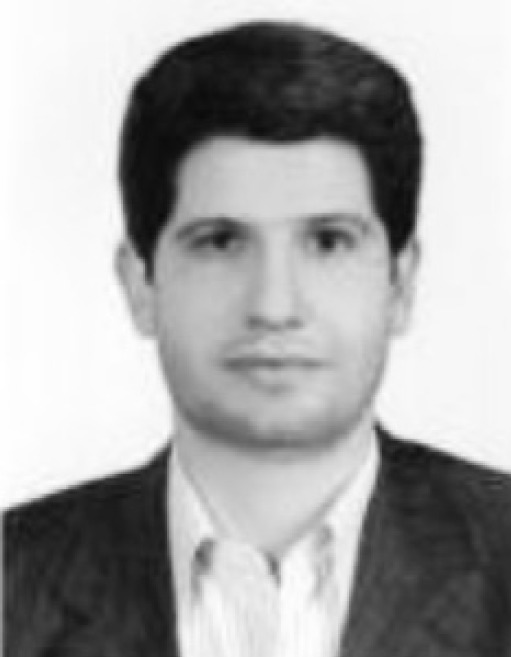


Competitiveness is considered as a key criterion for assessing the success of countries, industries and companies. World Economic Forum defines competitiveness as the set of institutions, policies and factors that determine the level of productivity. Moreover, competitiveness can be defined as acquisition more market share, greater profitability and long-term stability and growth of these indicators thereby improving the welfare and living standards of people. Putting it in perspective, companies and industries must be well competitive in domestic and international markets in order to survive. With respect to recent competitive and dynamic environment if companies want to be successful in competition arena, they must have competitive advantage which means creating and sustaining superior performance. The strength of competition depends on both the conduct of firms and the external business environment in which they compete, the state of infrastructure, legal framework and the effectiveness of the financial system. Mostly, barriers to competition in developing countries stemming from inappropriate government policies, and anti-competitive behavior of firms are common.

The healthcare systems across the world are constantly being subjected to differing needs of consumers. Hence, the supporting role of the pharmaceutical industry in developing and delivering good quality products to the society is critical to the success of the healthcare initiatives taken up by various public and private organizations. Pharmaceutical products account for 15 percent of global health spending and pharmaceutical cost per capita is $ 400 in high income countries and $ 4 in low income countries. Regarding that the large part of health care costs account for pharmaceutical expenditures, inefficiency of pharmaceutical production, procurement, storage and distribution are the most important part of disinvestment in health care. On the other hand, the social value of the pharmaceutical industry is apparent and undoubted. Not only it is the source of cost effective treatments which increase life expectancy and make better lives, but also it significantly contributes to the strength of the economy. Furthermore, modern medical advances would not be possible without the participation of the pharmaceutical industries in Research and Development.

The world pharmaceutical industry has been changing profoundly in the last decade. Intensive processes of concentration and consolidation have been continuing in all three sectors of the world pharmaceutical industry. Meanwhile, global competitiveness is becoming increasingly important for the pharmaceutical industry. Increased competitiveness and the changing structure of competitors impact the strategic direction of the world pharmaceutical companies in world. Competition is moderately profound since the pharmaceutical industry is still comparatively fragmented. Although the top 20 drug manufacturers control a little less than 60 percent of the market, no single company controls more than a 9 percent share. Competitors try to improve their position in the marketplace by means of price competition, acquisitions, advertising battles and new product introductions. This rivalry is particularly intense in saturated markets (*e.g.,* cardiovascular and central nervous systems, pain relievers), and less intense in growing markets (*e.g*., oncology or immune disorders).While competitiveness has been growing tremendously; there is an immediate requirement for the pharmaceutical companies to behave in an effective way.

In the twenty-first century, if pharmaceutical companies want to remain profitable, some steps need to take include; discovery of innovative medicines and marketing license obtaining as quickly as possible. Though the key to the sustainability of the industry is the continuous developments of new products which generate sufficient profitability and support the R&D spending of future products, pharmaceutical companies which want to be globally leading ones and successful business performers, need to primarily think entirely and differently about customers, markets and competitiveness. They also need to especially bear in mind that the needs of tomorrow’s customers are different from the needs of today’s customers. 

Though, managing pharmaceutical industry effectively and efficiently is vital in emerging countries for their health system and economy, according to the lack of economic motivations and low capacity of the government for covering the costs of innovative drugs in developing countries, the pharmaceutical industry usually doesn’t invest on novel drugs, and innovations are limited in such countries. Looking at an emerging market like Iran to enhance pharmaceutical industry` competitiveness, more focused should take on international collaboration with the large players in order to gain access to new products, know-how and quality control procedures in order to acquire skills. Such a strategy would then eventually lead to own investments and product development. Furthermore, policy makers in Iran should be aware about challenging they will face after being a member of World Trade Organization (WTO), albeit it needs to short and long term planning as well as using valuable experiences of countries like India, Jordan, and Turkey and so on. The importance of competitive advantage would be more critical and vital when nations have to compete in framework of WTO with the less tariff and duties. Summary, if our pharmaceutical companies want to perform and survive in global market, the culture of doing business should be profoundly changed from static orientation to dynamic one and think globally. 

*Gholamhossein Mehralian is currently working as a assistance at the Department of Pharmacoeconomic and Pharma management, School of Pharmacy, Shahid Beheshti University of Medical Sciences, Tehran, Iran. He could be reached at the following e-mail address:* gmehralian@gmail.com

